# Prehabilitation as a Strategy to Modulate the Tumor Microenvironment in Pancreatic Cancer

**DOI:** 10.3390/ijms27198900

**Published:** 2026-10-07

**Authors:** Sanjna Sandeep, Jason Schutt, Kelvin Allenson, Renee Stubbins, Junjun Zheng, Shu-Hsia Chen, Atiya Dhala

**Affiliations:** 1 Texas A&M School of Engineering Medicine, Texas A&M University, Houston, TX 77030, USA; sanjna0720@tamu.edu (S.S.); jschutt21@tamu.edu (J.S.); 2 Department of Surgery, Houston Methodist Hospital, Houston, TX 77030, USA; 3 Houston Methodist Neal Cancer Center, Houston, TX 77030, USA; restubbins@houstonmethodist.org; 4 Immunomonitoring Core, Houston Methodist Neal Cancer Center, Houston, TX 77030, USA; jzheng2@houstonmethodist.org (J.Z.); schen3@houstonmethodist.org (S.-H.C.); 5 Department of Surgery, J.C. Walter Jr. Transplant Center, Center for Critical Care, Houston Methodist Hospital, Houston, TX 77030, USA; afdhala@houstonmethodist.org

**Keywords:** pancreatic ductal adenocarcinoma, tumor microenvironment, multimodal prehabilitation, spatial immune profiling, imaging mass cytometry

## Abstract

Multimodal prehabilitation aims to enhance resilience to cancer therapy through exercise, nutrition, and psychological optimization. While prehabilitation can improve physical condition and functional status, its biologic influence on the tumor microenvironment (TME) remains poorly characterized. In pancreatic ductal adenocarcinoma (PDAC), the TME is dominated by immunosuppressive and desmoplastic features that limit immune cell infiltration and therapeutic efficacy. As an exploratory pilot analysis, this study characterized the cellular composition, spatial relationships, and functional signaling of the pancreatic TME in patients who underwent prehabilitation prior to surgical resection and those who did not. Tumor samples from eight patients (four prehabilitation, four control) were analyzed with a 34-plex protein panel using Imaging Mass Cytometry (IMC), categorizing the tumor–immune landscape and functional status at the single-cell resolution. Prehabilitation tumors demonstrated reduced neutrophil and epithelial/tumor cell density and enrichment of Natural Killer (NK), CD4^+^, and CD8^+^ T cells. Spatial analysis revealed an immune-permissive architecture in prehabilitation tumors, in contrast to immune-resistant patterns in controls. Functional marker analysis showed reduced activation and pro-tumor signaling in immunosuppressive populations, including Tregs (HLADR, pAkt, pNFκB) and Collagen^+^ CAFs (NOS2, pERK, pNFκB, pSTAT3). These preliminary findings raise the possibility that prehabilitation is associated with an immune-permissive TME in PDAC, supporting further investigation of its immunomodulatory potential in larger, prospectively designed cohorts.

## 1. Introduction

Pancreatic cancer is a lethal disease with a poor prognosis. According to the National Cancer Institute’s 2024 data, it is the third leading cause of cancer-related death in the United States despite ranking eleventh in incidence [1]. The most common histologic subtype, pancreatic adenocarcinoma (PDAC), arises from the exocrine pancreas and accounts for over 90% of pancreatic malignancies [2,3]. The etiopathogenesis of PDAC is multifactorial, involving a complex interplay between genetic mutations and environmental risk factors.

Frequent mutations in *KRAS*, *CDKN2A*, *TP53*, and *SMAD4* drive oncogenic signaling, metabolic reprogramming, and resistance to apoptosis, contributing to the disease’s aggressive biology and the limited efficacy of chemotherapy [3,4]. Additional risk factors include male sex, smoking, obesity, red meat consumption, and family history of pancreatic cancer [4].

Because early-stage disease is largely asymptomatic and effective screening tools are lacking, most patients present with advanced or metastatic disease [4]. CA19-9, the most commonly used biomarker, lacks sensitivity and specificity, as it is elevated in only approximately 65% of PDAC cases and may also rise in benign conditions such as cholestasis [4]. Consequently, fewer than 20% of patients are eligible for surgical resection at diagnosis, which is generally considered the only potentially curative therapy [2]. Even amongst those who complete all intended oncologic therapy, overall survival remains low, and there are few long-term survivors.

To improve perioperative surgical outcomes and resilience to treatment, prehabilitation has emerged as a promising intervention. Prehabilitation encompasses individualized programs that integrate exercise, nutritional optimization, and psychological support prior to surgery [4,5]. Several studies have demonstrated that such interventions can improve postoperative recovery, quality of life, and even long-term survival across various cancer types [6,7]. In pancreatic cancer, exercise-based prehabilitation has been associated with improvements in aerobic capacity and physical function, and emerging data suggest reductions in selective postoperative complications [8,9,10].

While prehabilitation emerged as a strategy to recover medically frail patients, defined as those with reduced functional capacity and diminished physiologic reserve, who reach surgical candidacy, previous evidence has suggested that prehabilitation may also influence tumor biology by increasing vascularity and enhancing antitumor immune activity [11,12]. The cellular and molecular mechanisms underlying these changes, particularly within the TME, remain poorly understood, and even when described, they have not proven vulnerable to therapeutic intervention.

The TME of PDAC is uniquely hostile and plays a central role in therapeutic resistance. It is characterized by a dense desmoplastic stroma, extensive extracellular matrix (ECM) deposition, and an abundance of immunosuppressive cells, such as tumor-associated neutrophils, myeloid-derived suppressor cells, and regulatory T cells (CD4Treg) [13]. Pancreatic stellate cells drive the formation of a fibrotic capsule around tumor nests, while elevated hyaluronan and matrix metalloproteinases (MMPs) further restrict immune cell infiltration and promote local invasion [13]. Even immune cells that penetrate such an environment appear senescent [13,14]. These interactions collectively establish an immune-excluded microenvironment in which cytotoxic T cells are physically and biochemically prevented from accessing malignant cells.

Understanding the PDAC tumor microenvironment is critical for developing effective prehabilitation strategies, as its dense stroma and immunosuppressive signaling can profoundly influence therapeutic response, surgical outcomes, and patient resilience [11]. Currently, evidence directly linking prehabilitation to tumor-level biological changes remains limited, with most mechanistic insights derived from preclinical models demonstrating that exercise can enhance tumor vascular structure, reduce hypoxia, augment Natural Killer (NK) and CD8^+^ T-cell infiltration, and improve chemotherapy efficacy [11,15,16]. In pancreatic cancer, early translational work using patient-derived xenograft mouse models demonstrated that structured exercise during neoadjuvant therapy increased tumor vascularity and had more open microvessel lumens compared with non-exercising controls [15]. Building upon this rationale, this exploratory pilot study aims to characterize the cellular composition, spatial architecture, and functional signaling of the PDAC TME in patients who underwent a formal prehabilitation program compared with those who did not. Using Imaging Mass Cytometry (IMC), this study employs a 34-plex marker panel to profile cellular phenotypes and tumor–immune spatial interactions comprehensively. This analysis seeks to explore the potential mechanistic benefits of prehabilitation at the cellular and immunologic levels in pancreatic cancer.

## 2. Results

### 2.1. Cellular Composition

**Figure 1:**
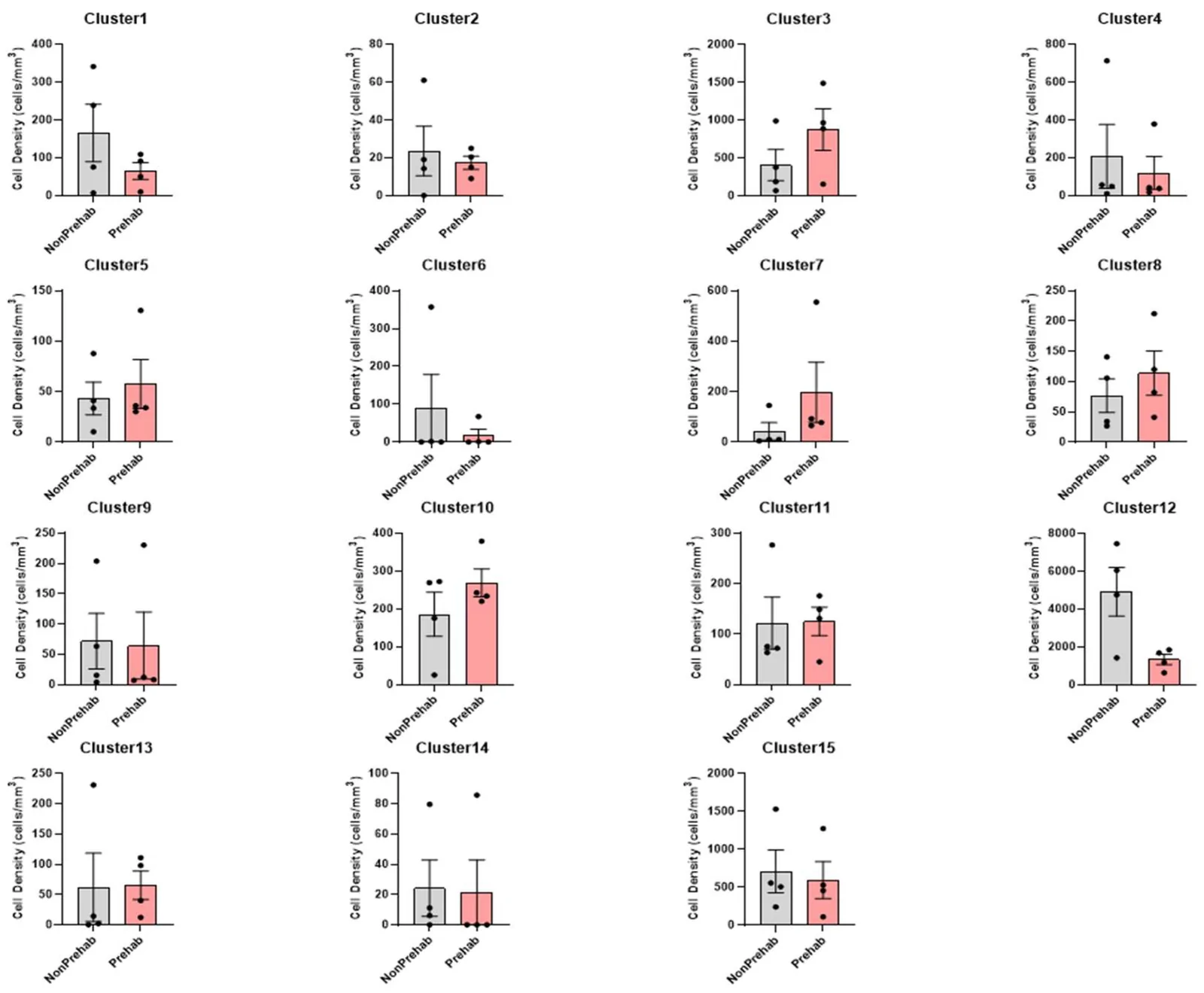
Cell densities. Densities of fifteen identified cell clusters in prehabilitation and non-prehabilitation tumors, normalized to region-of-interest area. Each panel shows mean density with variability for a specific cluster. Bars show mean ± standard error of the mean (SEM), and dots represent individual patients (n = 4 per group). Grey, non-prehabilitation; red, prehabilitation.

**Figure 2:**
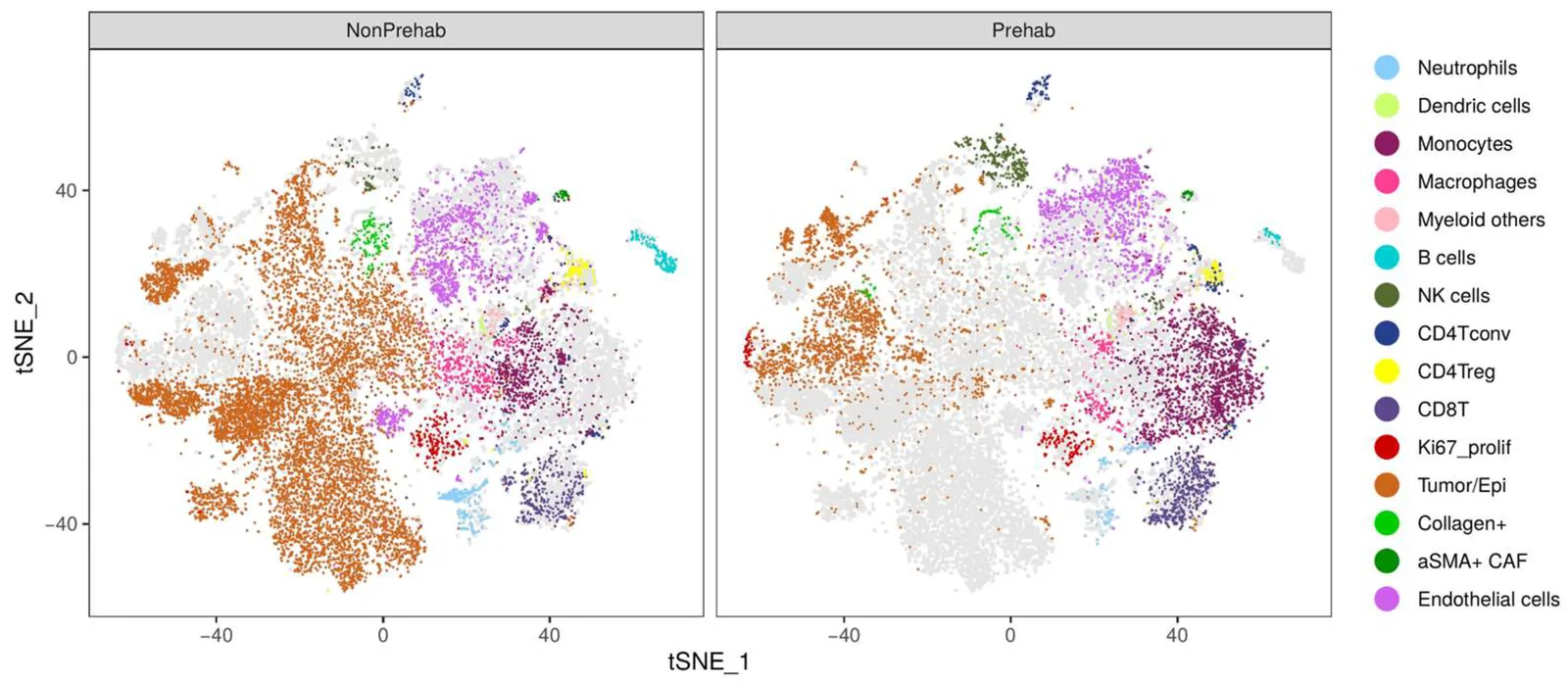
t-distributed stochastic neighbor embedding (t-SNE) plots showing single-cell distributions for the fifteen clusters in representative non-prehabilitation and prehabilitation tumors. Colors correspond to annotated epithelial, immune, and stromal cell populations. The plots demonstrate clear segregation of epithelial, immune, and stromal populations and highlight differences in relative immune composition between groups.

Imaging mass cytometry identified fifteen cell clusters representing epithelial, immune, and stromal populations (Table 1). Clusters were annotated using canonical markers, including CD15 for neutrophils, CD68 for macrophages, CD4 and CD8 for lymphocytes, alpha-SMA for fibroblasts, and CD31 for endothelial cells. When normalized to the region-of-interest (ROI) area, prehabilitation tumors trended toward lower mean densities of neutrophils (Cluster 1) and epithelial/tumor cells (Cluster 12) relative to the non-prehabilitation group. In contrast, NK cells (Cluster 7), CD4^+^ conventional T cells (CD4Tconv) (Cluster 8), and CD8^+^ cytotoxic T cells (Cluster 10) trended higher in the prehabilitation cohort. These differences were observed across randomly sampled regions and were not attributable to variation in ROI size (Figure 1). Two-dimensional t-distributed stochastic neighbor embedding (t-SNE) plots demonstrated consistent separation of epithelial, lymphoid, myeloid, and stromal compartments across all specimens (Figure 2).

### 2.2. Spatial Neighborhood Analysis

**Figure 3:**
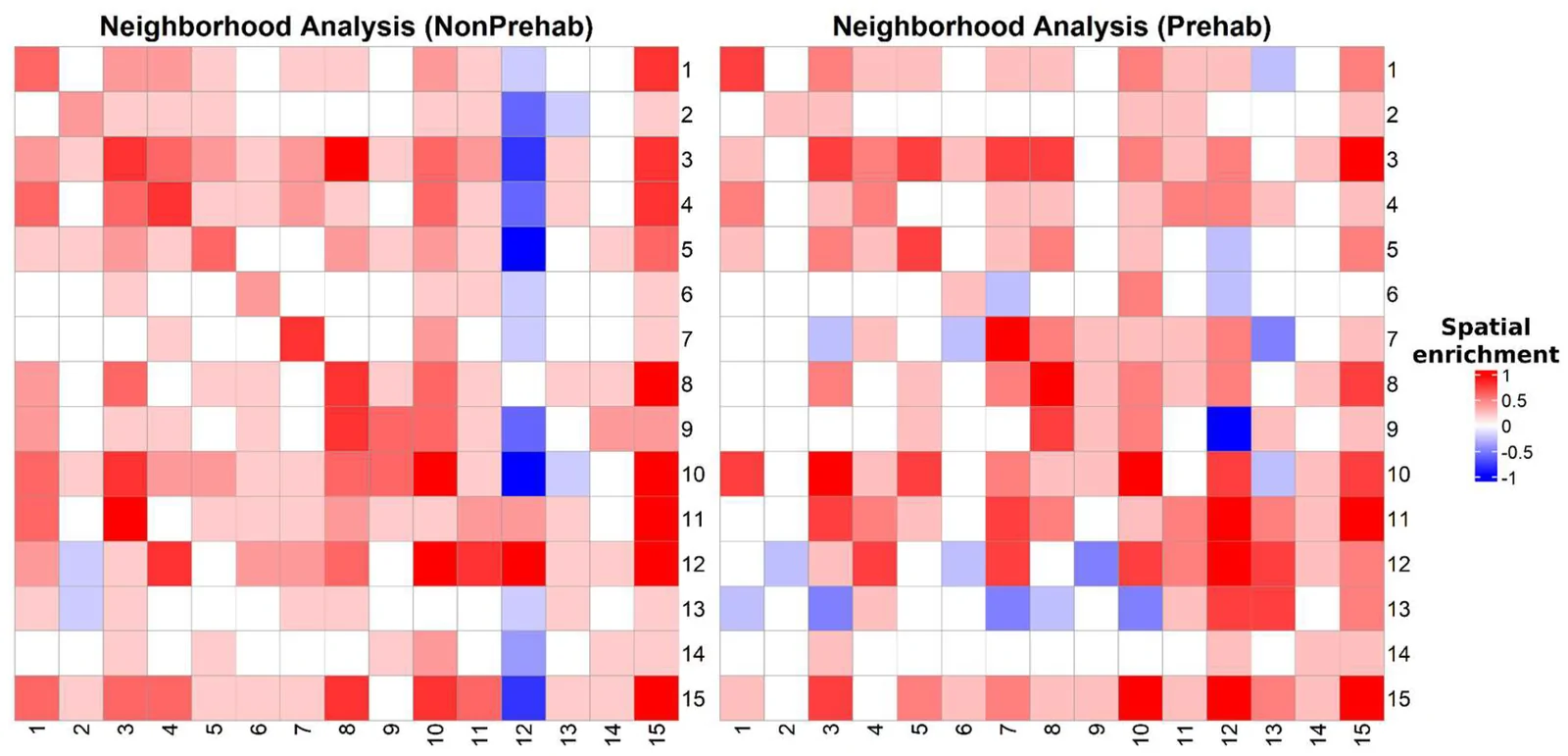
Neighborhood Analysis Heatmaps. Heatmaps showing averaged neighborhood spatial enrichment values for all pairwise combinations of the fifteen cell clusters. Positive scores indicate enriched proximity, and negative scores indicate spatial avoidance. Non-prehabilitation tumors display depleted interactions between epithelial/tumor clusters and immune subsets, whereas prehabilitation tumors exhibit enriched epithelial–immune spatial proximity.

**Figure 4:**
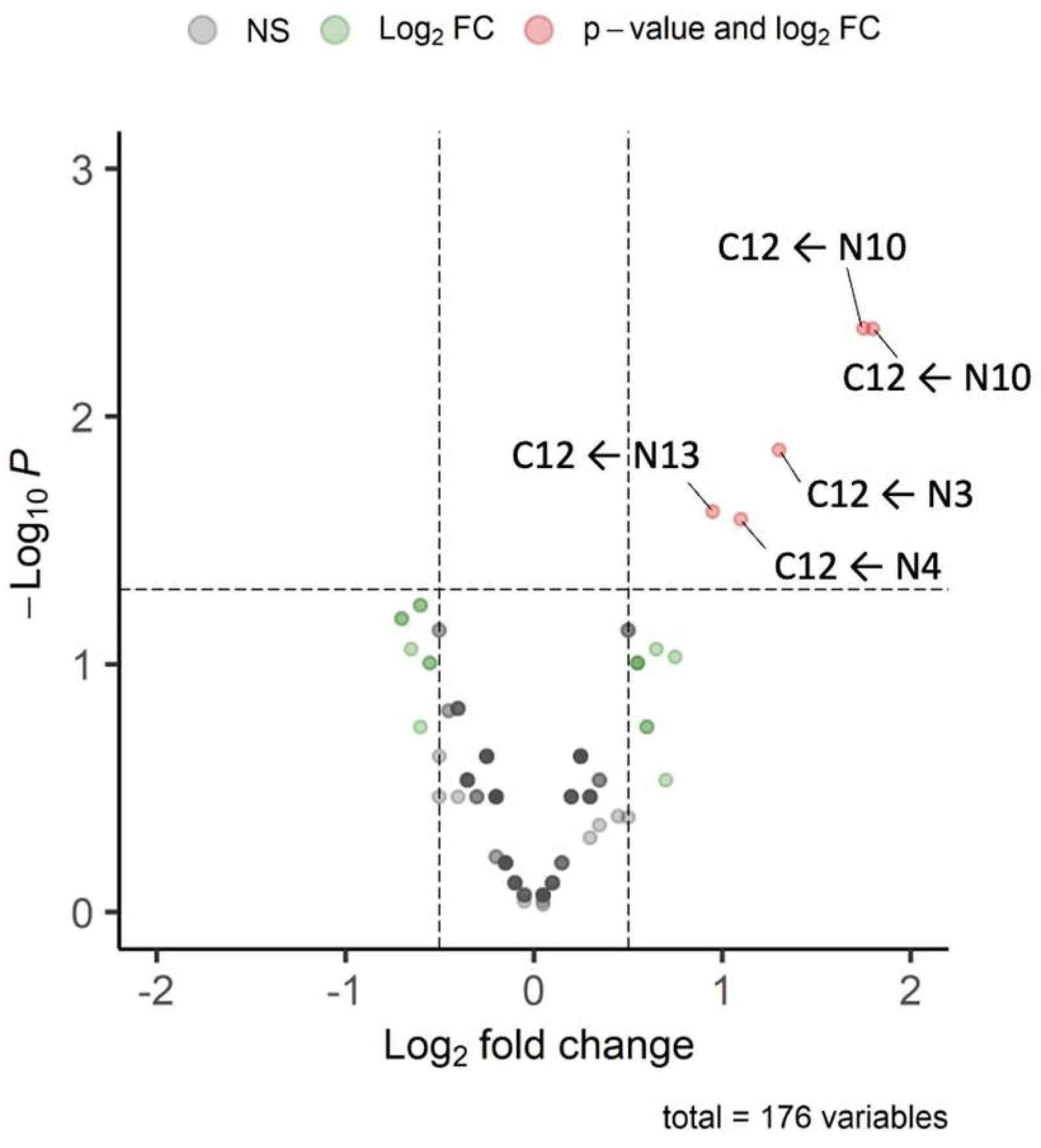
Differential Cluster–Neighbor Interaction Frequencies in Prehabilitation versus Control. For each of the 176 tested interactions, “Center” (C) refers to the focal cell cluster and “Neighbor” (N) refers to the adjacent cluster type, as defined by imaging mass cytometry-based cluster phenotyping (Table 1). Volcano plot comparing 176 cluster–neighbor interaction frequencies between groups. Points represent log_2_ interaction differences and statistical significance, highlighting altered epithelial–immune and epithelial–stromal proximities. Positive log_2_ fold change values indicate higher interaction frequency in the prehabilitation cohort. Grey points, not significant; green points, |log_2_ fold change| > 0.5 only; red points, *p* < 0.05 and |log_2_ fold change| > 0.5. Dashed lines mark log_2_ fold change = ±0.5 and *p* = 0.05.

Spatial neighborhood analysis revealed distinct cell–cell proximity patterns between groups (Figure 3). In the non-prehabilitation cohort, tumor/epithelial cells (Cluster 12) showed negative spatial enrichment for interactions with neutrophils (Cluster 1) and collagen-producing fibroblasts (Cluster 13), indicating depleted spatial associations. In contrast, prehabilitation tumors demonstrated positive spatial enrichment for interactions between tumor/epithelial cells (Cluster 12) and NK cells (Cluster 7), CD8^+^ T cells (Cluster 10), and endothelial cells (Cluster 15). Given the small sample size, FDR-corrected significance was not retained for any of the 176 tested interactions (q ≈ 0.39–0.59); however, an exact leave-one-out permutation test identified several interactions, most notably increased epithelial/tumor cell (Cluster 12) proximity to endothelial cells (Cluster 15) and CD8^+^ T cells (Cluster 10), that persisted as robust signals even after excluding the neoadjuvant-treated patient (Figure 4, Table 2).

### 2.3. IMC Validation

**Figure 5:**
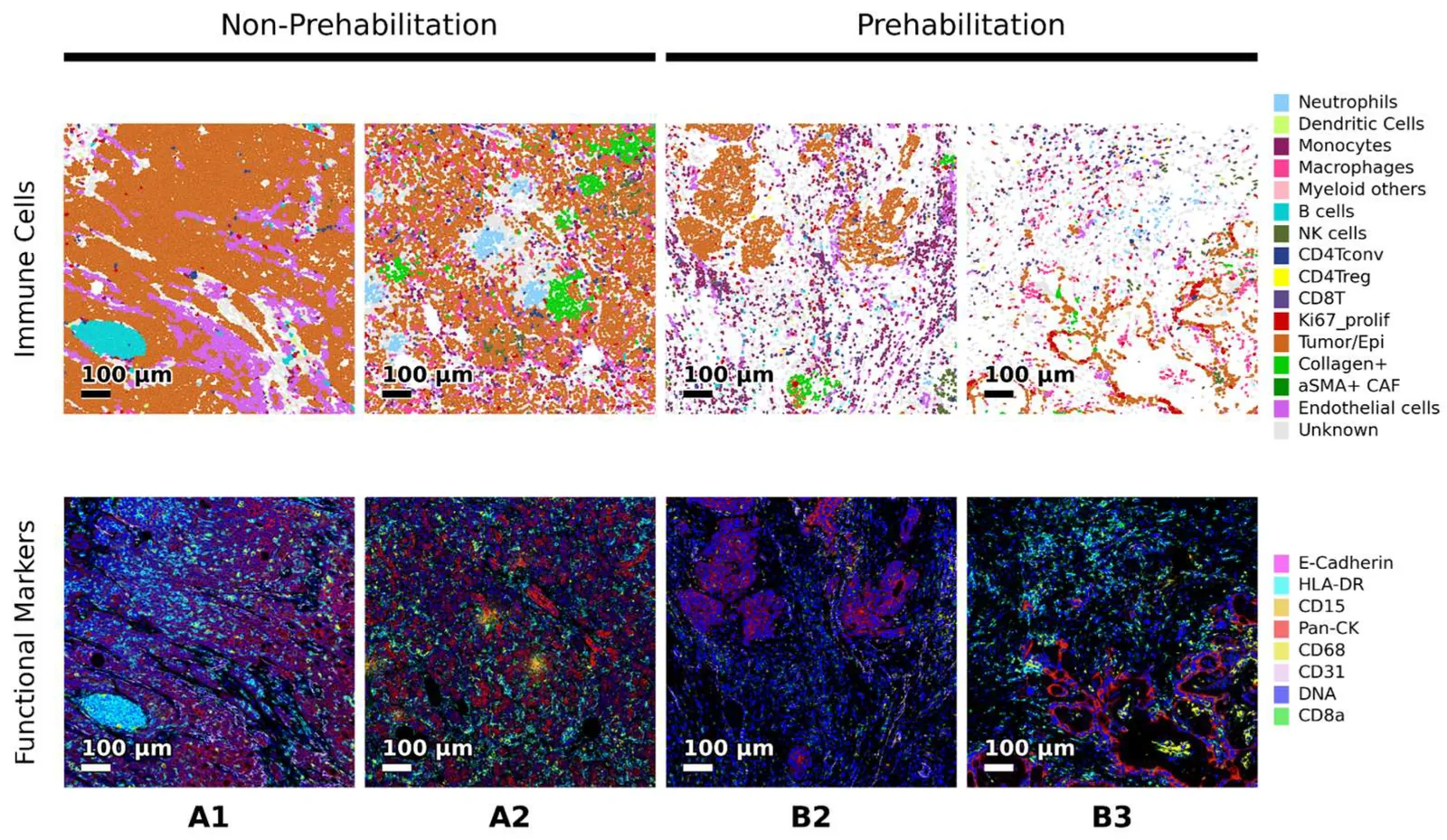
IMC staining of the pancreatic TME. Representative IMC segmentation and immunohistochemistry overlays from non-prehabilitation (A1, A2) and prehabilitation (B2, B3) tumors, corresponding to the patient profiles listed in Table 3. Markers include E-cadherin, Pan-CK, CD15, CD68, CD8, HLA-DR, and CD31. Non-prehabilitation tumors show continuous epithelial regions with concentrated myeloid cells, whereas prehabilitation tumors display fragmented epithelium with more dispersed immune-cell distribution.

In the non-prehabilitation group, IMC segmentation (Figure 5) shows large, continuous regions of epithelial or tumor cells, with surrounding stromal areas containing alpha-SMA-positive fibroblasts and CD15^+^ and CD68^+^ myeloid cells. HLA-DR signal is concentrated in the peritumoral stroma and frequently overlaps with CD15 and CD68. CD8^+^ lymphocytes are primarily located at the tumor–stroma interface with limited presence within epithelial regions. CD31 staining outlines vascular structures at the periphery of epithelial clusters.

In the prehabilitation group, epithelial or tumor cell regions appear smaller and more fragmented, with intervening immune cell populations. CD8^+^ lymphocytes are present in both stromal and epithelial areas. CD15^+^ and CD68^+^ cells appear less compactly organized around epithelial structures compared with the non-prehabilitation samples. The HLA-DR signal is more diffuse across stromal regions, and CD31 staining delineates vascular structures with a distribution similar to that of the non-prehabilitation group.

**Table 3:** Cohort demographics. Baseline characteristics of the eight patients included in the study stratified by prehabilitation status. Variables include age, sex, race, BMI, tumor differentiation, tumor size, lymph node (LN) involvement, total lymph nodes examined, and type of pancreatic resection performed.

Patient ID	Age	Sex	Race	BMI	Differentiation	Tumor Size	LN Positive	LN Total	Surgery
**Non-Prehabilitation Cohort**									
A1	54	M	Caucasian	31.89	Poor	3.5	5	14	Whipple
A2	77	M	Caucasian	22.96	Moderate to Poor	3.5	12	22	Distal Pancreatectomy
A3	72	F	Hispanic or Latino	26.66	Poor	2.5	5	9	Whipple
A4	76	F	Caucasian	25.00	Well to moderate	3.8	0	23	Whipple
**Prehabilitation Cohort**									
B1	75	F	Black	42.87	Well	3.2	3	22	Distal Pancreatectomy
B2	69	M	Caucasian	22.96	Poor	5	1	13	Distal Pancreatectomy
B3	75	F	Black	18.01	Moderate	2.5	0	22	Whipple
B4	63	F	Caucasian	27.98	Moderate	1.6	0	16	Whipple

### 2.4. Functional Marker Expression

**Figure 6:**
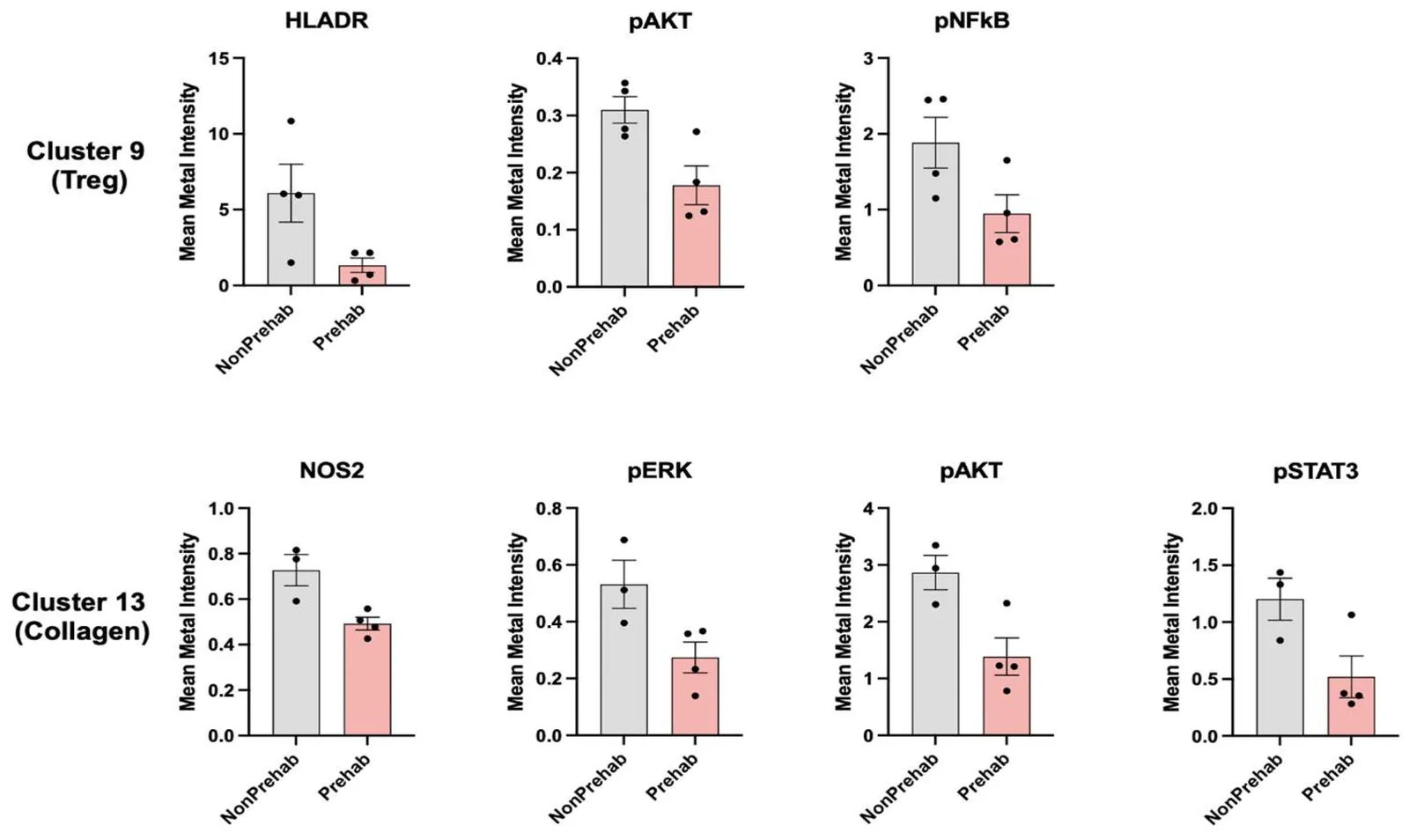
Functional Marker Intensity Plot. Normalized intensity values for functional signaling markers (HLADR, pAkt, pNFκB, NOS2, pERK, pSTAT3) across immune and stromal subsets in prehabilitation and non-prehabilitation tumors. Panels highlight down-regulation of inflammatory and protumorigenic signaling within regulatory T cells and Collagen^+^ CAFs in the prehabilitation cohort.

Functional marker analysis showed consistent attenuation of inflammatory and protumorigenic signaling pathways in the prehabilitation cohort (Figure 6). Regulatory T cells (Cluster 9) displayed lower expression of HLADR, pAkt, and pNFκB relative to controls. Collagen-positive fibroblasts (Cluster 13) exhibited reduced NOS2, pERK, pNFκB, and pSTAT3 expression. Groupwise statistical testing showed lower expressions of NOS2 (*p* = 0.0166), pERK (*p* = 0.0424), pNFκB (*p* = 0.0246), and pSTAT3 (*p* = 0.0499) in prehabilitation tumors. These findings were reproducible across ROIs and are consistent with decreased inflammatory activation and a less suppressive stromal phenotype.

## 3. Discussion

This exploratory pilot study evaluated the TME of patients with PDAC who completed multimodal prehabilitation before tumor resection. Across imaging, mass cytometry, spatial analysis, and functional profiling, the TMEs of those who underwent prehabilitation were linked to lower epithelial and tumor cell densities, reduced neutrophil representation, and higher NK cell, CD4^+^ T cell, and CD8^+^ T cell densities. Spatial proximity analysis further showed that the prehabilitation cohort exhibited enriched interactions between epithelial cells and cytotoxic lymphoid populations. In contrast, the TMEs from patients who did not undergo prehabilitation demonstrated enriched interactions between epithelial regions and stromal or myeloid cells. Functional marker expression was also lower in the prehabilitation cohort, with reduced levels of HLADR, pAkt, pNFκB, pERK, pSTAT3, and NOS2 in regulatory T cells and collagen-producing stromal populations. These patterns were consistent across multiple regions of interest and could possibly suggest reproducible differences in intratumoral immune composition, spatial organization, and inflammatory signaling associated with prehabilitation.

The companion study reported findings that largely converge with ours, including reduced fibroblast and TGF-β signaling that parallels our CAF functional marker data [17]. Neutrophils were an exception, as that study found higher MPO^+^ density and neutrophil-associated transcriptional signatures with prehabilitation, whereas our CD15^+^ IMC density was lower. We attribute this to differences in tissue sampling and CD15 marker behavior, since CD15 can be shed during neutrophil activation while MPO persists, rather than to a true biological conflict.

Clinically, multimodal prehabilitation has been associated with improvements in aerobic capacity, nutritional status, frailty metrics, and postoperative outcomes across diverse cancer populations [18,19]. In pancreatic surgery, several studies have demonstrated the feasibility of prehabilitation and reported improvements in functional reserve prior to resection [20,21]. To date, these benefits have been mainly evaluated in the context of perioperative outcomes rather than tumor biology. However, emerging evidence suggests that physiologic conditioning may influence tumor-adjacent immune features. In a randomized trial of prehabilitation administered during neoadjuvant therapy for esophageal adenocarcinoma, Rayner et al. observed increased intratumoral CD8^+^ and CD56^+^ NK-cell infiltration and the development of more mature tertiary lymphoid structures, despite no clear differences in early oncologic endpoints [22].

The intratumoral differences observed in this study align with established knowledge of PDAC biology. Prior studies have demonstrated that the PDAC microenvironment contains prominent populations of fibroblasts, myeloid cells, and extracellular matrix components that limit immune cell access to tumor epithelium [23,24,25]. Spatial transcriptomic analyses further indicate that the proximity of cytotoxic lymphocytes to epithelial cells, rather than total immune density, is associated with improved outcomes [26,27]. As a result, research has suggested that future studies should focus on modulating the TME to enhance the therapeutic efficacy of current therapies [28]. The neighborhood analysis presented in the current study suggests that the TMEs of patients who did not undergo prehabilitation showed markedly reduced epithelial–lymphocyte interactions, consistent with an immune-excluded tumor microenvironment. In contrast, the TMEs in patients who had undergone prehabilitation exhibited increased spatial proximity between tumor epithelium and both NK cells and CD8^+^ T cells, possibly indicating a shift toward a more favorable immune phenotype.

Prior studies in pancreatic ductal adenocarcinoma have reported that FOXP3-positive regulatory T cells accumulate during the progression from precursor lesions to invasive carcinoma and are associated with adverse histopathologic features and reduced survival [29,30,31]. These findings emphasize that the functional state of Tregs, rather than their absolute abundance, contributes to the immunosuppressive milieu characteristic of PDAC. In the present study, we did not observe reductions in overall Treg or CAF population densities, and no statistically significant decreases in additional functional markers were detected within the epithelial/tumor cells (C12) beyond the reduced cell density already reported.

Instead, the most reproducible differences associated with prehabilitation were dampened activation and inflammatory signaling within Tregs and CAFs, together with increased spatial proximity of NK and CD8^+^ T cells to epithelial regions. Within the scope of this study, although no clinical inferences can be made, the findings suggest that prehabilitation may possibly alter immune and stromal functional states, potentially influencing epithelial–lymphocyte interactions and local immune accessibility. Larger studies incorporating clinical tumor metrics, additional functional markers, and longitudinal sampling will be needed to determine whether these patterns translate into measurable differences in tumor burden or clinical outcomes.

Exercise-based prehabilitation has generally been indicated and evaluated for its beneficial effects on patient functional status and perioperative surgical outcomes, rather than as a means to augment the therapeutic efficacy of cancer-directed therapies on the TME. A recent randomized trial in esophageal adenocarcinoma demonstrated that prehabilitation during neoadjuvant chemotherapy increased intratumoral CD8 and CD56-positive NK-cell infiltration and promoted the development of more mature tertiary lymphoid structures, without clear short-term differences in clinical outcomes [22]. These findings support the concept that physiologic conditioning can remodel the tumor immune microenvironment even when the bulk tumor burden remains similar. Analogously, in our study, prehabilitation was associated with reduced expression of NOS2, pSTAT3, pERK, and pNFκB within collagen-rich stromal regions and within Tregs. Preclinical work in pancreatic cancer has implicated STAT3 activation in cancer-associated fibroblasts as a driver of an immunosuppressive stroma that limits CD8 T-cell infiltration and accelerates tumor progression [32]. Our observation that patients who underwent prehabilitation had lower stromal pSTAT3 and other stress-response markers, together with greater NK and CD8 cell proximity, raises the hypothesis that prehabilitation may partially recapitulate the effects of pharmacologic stromal modulation by creating a more immune-permissive microenvironment. These data, while preliminary, provide a strong rationale for future studies integrating radiographic or volumetric tumor size measures, functional immune markers, and longitudinal changes in cancer metabolomics. The interplay between neoadjuvant therapies, prehabilitation, and clinical outcomes will be complex, but it is a topic for future study.

Potential mechanisms linking prehabilitation to these intratumoral differences are supported by prior exercise-oncology and immunometabolic studies. Aerobic exercise mobilizes IL-6-dependent NK-cell populations and enhances IL-15-mediated CD8^+^ T-cell activation, increasing antitumor immune activity and slowing tumor progression in several models [16,33]. In murine PDAC, structured aerobic training increases vascular perfusion, promotes CD8^+^ infiltration, and improves responses to immune checkpoint blockade [16,34]. Nutritional interventions can also reduce systemic inflammation and improve lymphocyte competence, particularly in settings of protein deficiency, sarcopenia, or hypoalbuminemia [13,20,21]. Reduced circulating IL-6 and TNF-α following nutritional repletion may attenuate fibroblast activation and matrix deposition [13]. Psychosocial interventions may decrease sympathetic signaling, physiologic stress-mediated glucocorticoid immunosuppression, and myeloid bias [12]. Although systemic measurements were not collected in this study, the observed intratumoral patterns are consistent with these previously described physiologic effects.

Stromal signaling differences observed here also correspond to CAF-driven immune-exclusion pathways. STAT3-, NFκB-, iNOS-, and ERK-mediated signaling in fibroblasts have been associated with extracellular matrix remodeling, inflammatory cytokine secretion, and recruitment of immunosuppressive myeloid populations in PDAC [13,24]. Pharmacologic inhibition of these pathways, including FAK inhibition, CXCL12/CXCR4 blockade, and CD40-mediated macrophage activation, enhances CD8^+^ infiltration and reduces stromal barriers [13,35,36,37]. The decreased expression of NOS2, pERK, pNFκB, and pSTAT3 in prehabilitation TME reflects a signaling pattern consistent with lower fibroblast activation and reduced stromal immunosuppression.

These exploratory findings provide preliminary evidence that multimodal physiologic conditioning may be associated with differences in tumor immune composition, spatial accessibility, and inflammatory signaling at the time of resection. These observations may have implications for both surgical and oncologic care. A more immune-accessible TME at the time of surgery may influence postoperative recovery, the efficacy of adjuvant therapy, or the success of emerging systemic therapies, including immune therapies.

This study has several limitations. The sample size was small, comprising only eight patients in total, which limits generalizability and increases the potential for a Type II error. Because of the study design and the exploratory nature of this study, cohort demographics could not always be adequately matched. Notably, BMI ranged widely across both groups (18.0–42.9), tumor differentiation spanned well to poor, and nodal involvement ranged from 0 to 12 positive lymph nodes. These baseline differences may independently influence systemic inflammation and the TME, independent of prehabilitation status. The retrospective design also further restricts causal inference. Patients were not randomized, and participation in prehabilitation may reflect unmeasured differences in baseline fitness, comorbidities, or social factors that could influence the tumor microenvironment. One patient in the prehabilitation cohort received neoadjuvant chemotherapy, which could independently affect immune and stromal composition. A sensitivity analysis excluding this patient confirmed that the primary directionality of observed differences remained consistent, suggesting that findings were not driven by this individual case. Differences in tumor differentiation were present between cohorts and may also contribute to the observed findings. Finally, the study focused on histologic and immunologic features, but did not assess clinical endpoints, such as postoperative recovery, complications, or survival. Larger and prospectively matched cohorts that incorporate clinical outcomes will be essential to validate these preliminary observations.

Future studies should evaluate the biological effects of prehabilitation in larger, prospectively enrolled patient cohorts. The integration of spatial proteomics, single-cell transcriptomics, and metabolomic profiling will be necessary to define the mechanisms by which exercise, nutritional conditioning, and psychosocial optimization influence systemic inflammation, vascular perfusion, myeloid skewing, and intratumoral immune dynamics. A critical next step is to correlate these biologic changes with clinical endpoints, including postoperative recovery, recurrence-free survival, and response to adjuvant therapy. These studies should also include standardized definitions and adherence metrics for prehabilitation to improve reproducibility across institutions. Establishing these relationships will clarify whether prehabilitation provides meaningful oncologic benefits in addition to functional improvements. Ultimately, this work may potentially support the development of biomarker-guided and personalized prehabilitation protocols that optimize both host physiology and tumor immunobiology, thereby improving surgical and oncologic outcomes in pancreatic cancer.

## 4. Materials and Methods

### 4.1. Patient Cohort

This retrospective exploratory pilot study included surgical resection specimens from eight patients with histologically confirmed pancreatic ductal adenocarcinoma (PDAC) who underwent pancreatectomy at Houston Methodist Hospital from 2019 to 2024. All patients provided written informed consent under IRB-approved tissue-use protocols. Four patients completed a multimodal prehabilitation program prior to surgery, and four matched controls underwent standard preoperative management without formal prehabilitation.

Patients were selected based on (1) availability of adequate resection tissue for imaging mass cytometry (IMC), (2) confirmation of PDAC on final pathology, and (3) documented prehabilitation participation status. Matching considerations included age, sex, BMI, tumor grade, and resection type when feasible. However, given the limited pool of eligible patients and the exploratory nature of this study, complete matching was not achievable and notable baseline differences in BMI, tumor differentiation, and nodal status remained between cohorts (Table 3).

### 4.2. Prehabilitation Protocol

Patients with histologically confirmed pancreatic cancer evaluated in surgical or oncology clinics were screened using a Houston Methodist Hospital-specific frailty index assessing fatigue, functional capacity, comorbidities, and recent weight change. Patients with resectable or borderline resectable disease who met eligibility criteria were enrolled in the institutional prehabilitation program. Prehabilitation duration was individualized and continued until patients were considered optimized for surgery. The multimodal program was implemented before surgical resection and included components for nutrition, physical therapy, respiratory care, and psychological support. Nutritional prehabilitation consisted of a dietitian-led assessment and counseling session, conducted in person or remotely, with formal screening using the Scored Patient-Generated Subjective Global Assessment (PG-SGA). Counseling was individualized, with a target protein intake of approximately 1.5 g/kg/day when medically appropriate. Patients with PG-SGA scores greater than nine received at least three additional counseling sessions. Physical prehabilitation included aerobic and resistance training. Patients were instructed to walk daily for at least 20 min at a moderate intensity and to attend supervised physical therapy sessions 1 to 2 times per week. At-home resistance training using elastic bands was prescribed at least two days per week and progressed based on individual capacity. Respiratory conditioning was addressed through inspiratory muscle training. Baseline maximal inspiratory pressure was measured, and patients completed a progressive at-home training regimen consisting of two 5-min sessions on at least five days per week using a handheld inspiratory muscle trainer. Psychological support was provided through motivational interviewing and mindfulness-based interventions delivered by a trained prehabilitation coordinator. Program adherence and readiness for surgery were assessed through periodic follow-up evaluations.

### 4.3. Imaging Mass Cytometry (IMC)

Tissue sections (4 μm formalin-fixed, paraffin-embedded) were stained with a pathologist-verified panel of metal-conjugated antibodies optimized by the Houston Methodist ImmunoMonitoring Core. A 34-plex marker panel was deployed to cover both phenotypic markers of immune, epithelial, and stromal cells, as well as functional markers of cell signaling pathways. Metal labeling was performed according to Standard BioTools (South San Francisco, CA, USA) protocols, with absorbance measurements taken. Antibodies were stored in Candor PBS stabilization buffer (Candor Bioscience GmbH, Wangen im Allgäu, Germany) at 4 °C. Sections were baked, dewaxed in xylene, rehydrated through graded alcohols, and heat-induced epitope retrieval in Tris-Tween20 buffer (pH 9) at 95 °C for 20 min. After blocking with 3% BSA, slides were incubated overnight with the antibody master mix, washed, and counterstained with Cell-ID Intercalator (Standard BioTools) for nuclear identification. Subsequently, the slides were washed, air-dried, and stored for ablation. Following drying, samples were ablated using the Hyperion Imaging System (Standard BioTools) for acquisition.

### 4.4. Data Processing and Analysis

IMC data were preprocessed and checked for tissue integrity, staining quality, and signal range prior to analysis. Single cells were segmented using ilastik (https://www.ilastik.org) and CellProfiler (https://cellprofiler.org), guided by nuclear staining (Ir191) and other surface markers [38,39]. Following cell segmentation, data are processed using the Histology Topography Cytometry Analysis Toolbox (HistoCAT; https://github.com/BodenmillerGroup/histoCAT, accessed on 2 July 2026), in which the mean intensities of each marker for all single cells are extracted [40]. Data from all samples are consolidated in RStudio (version 2025.09.1, Posit Software, Boston, MA, USA; https://posit.co) scripts for downstream analysis. The intensity values for each marker are clipped at the 99.5th percentile to eliminate outliers and subsequently normalized to a 0 to 1 range, giving equal weights to each marker. The normalized intensities are used to perform unsupervised clustering in Seurat (https://satijalab.org/seurat, accessed on 2 July 2026) using the Louvain algorithm [41]. Cell clusters are annotated based on marker expression averages and consolidated into fifteen canonical classes of immune, stromal, and tumor cells. Cell densities of each cell type are calculated by normalizing cell counts to the corresponding ROI (region of interest) areas.

### 4.5. Spatial and Functional Analysis

Neighborhood analysis in HistoCAT identifies the statistically significant neighboring status for each pair of cell types [40]. For each patient, the neighborhood analysis categorizes the results (or the relationship) to −1 to 1, where 1 (or red) denotes that two cell types are neighboring, −1 (or blue) indicates they are significantly avoiding each other, and 0 indicates that no significant spatial relationship is observed between the corresponding cell types. The neighborhood heatmap (Figure 3) summarizes the pairwise neighboring status across conditions by averaging the quantified values of all samples within the conditions. Further, the Cochran–Armitage Trend Test is used to statistically compare the neighboring status changes across conditions [40,42]. Functional markers analyzed included HLADR, pAkt, pNFκB, NOS2, pERK, pSTAT3, and collagen, representing immune activation, tumor-driven inflammation, and stromal remodeling.

### 4.6. Statistical Analysis

Analyses were performed at the patient level, with regions of interest summarized within each specimen to avoid treating multiple regions from the same patient as independent observations. Regions of interest were collected across two data collection periods (2023 and 2025). Sample-level metadata reflecting collection period is provided in Table S1. Cell-type densities were calculated as cells per unit ROI area, and functional marker expression was summarized using normalized marker intensity values generated from the preprocessing pipeline. Groupwise comparisons between prehabilitation and non-prehabilitation cohorts were assessed using Student’s *t*-test. To further evaluate the robustness of these findings given the limited sample size, an exact leave-one-out (LOO) sample-label permutation test was performed. For the 176 pairwise spatial interactions evaluated (Figure 4), Benjamini–Hochberg FDR correction did not yield significant q-values (q ≈ 0.39–0.59), so results were additionally assessed using the LOO permutation approach as a robustness check (Table 2). A sensitivity analysis excluding the neoadjuvant-treated patient was also performed to confirm that observed differences were not driven by this individual. Given the exploratory nature of this pilot study and multiple comparisons across cell subsets, P values were interpreted descriptively.

## 5. Conclusions

In this exploratory pilot analysis, multimodal prehabilitation prior to pancreatectomy was associated with differences in the immune composition of the pancreatic tumor microenvironment. Tumors from the prehabilitation cohort trended toward reduced epithelial and tumor cell density; lower neutrophil representation; enrichment of NK, CD4^+^, and CD8^+^ T-cell populations; and spatial patterns pointing to greater immune and epithelial proximity. Functional profiling showed lower activation of inflammatory and stromal signaling pathways, including pNFκB, pSTAT3, pERK, and NOS2, possibly suggesting attenuation of tumor-driven inflammation and reduced fibroblast-mediated immune exclusion.

Although preliminary, these findings support the concept that systemic physiologic conditioning may possibly influence intratumoral biology in addition to improving functional reserve. Prehabilitation is safe, feasible, and widely scalable within contemporary surgical pathways. If validated in larger prospective cohorts, it may represent a non-pharmacologic strategy that enhances immune surveillance, promotes a more permissive tumor architecture, and augments responsiveness to cytotoxic or immune-based therapies in pancreatic ductal adenocarcinoma.

## Figures and Tables

**Table 1:** Cell cluster annotation and refinement table used for single-cell analysis. Marker-based classification of the fifteen single-cell clusters identified by Imaging Mass Cytometry.

Cluster	Markers	Cell Types	Major Categories
Cluster 1	CD15^+^	Neutrophils	Myeloid Cells
Cluster 2	CD1c^+^	Dendritic cells	
Cluster 3	CD14^+^ (CD163^+^)	Monocytes	
Cluster 4	HLADR^+^CD68^+^	Macrophages	
Cluster 5	HLADR^+^	Other myeloid cells	
Cluster 6	CD20^+^	B cells	Lymphocytes
Cluster 7	CD56^+^	NK cells	
Cluster 8	CD4^+^Foxp3^−^	CD4Tconv	
Cluster 9	CD4^+^Foxp3^+^	CD4Treg	
Cluster 10	CD8^+^(GranZB^+^)(Tbet^+^)	CD8T cells	
Cluster 11	Ki67^+^	Prolif	Epi or Tumor Cells
Cluster 12	Ecad^+^/PanCK^+^/PD-L1^+^	Epi/Tumor	
Cluster 13	Collagen^+^	Collagen^+^ CAF	Stromal Cells
Cluster 14	aSMA^+^	aSMA^+^ CAF	
Cluster 15	CD31^+^	Endothelial cells	

**Table 2:** Spatial interaction robustness under FDR correction and leave-one-out permutation testing. For the top five cluster–neighbor interactions by nominal *p*-value (Figure 4), FDR-adjusted q-values did not reach significance at this sample size; an exact leave-one-out (LOO) sample-label permutation test was used as a robustness check.

Interaction	Nominal *p*	FDR q	LOO Perm. *p*
C12 ← N10	0.0044	0.39	0.008
C12 ← N15	0.0044	0.39	0.016
C12 ← N3	0.0136	0.55	0.032
C12 ← N13	0.0243	0.55	0.040
C12 ← N4	0.0261	0.55	0.079

## Data Availability

The data presented in this study are available on request from the corresponding author due to restrictions related to patient privacy and institutional ethical considerations.

## Supplementary Materials

The following supporting information can be downloaded at https://www.mdpi.com/article/10.3390/ijms27198900/s1.

## Abbreviations

The following abbreviations are used in this manuscript:

TME	Tumor microenvironment
PDAC	Pancreatic ductal adenocarcinoma
IMC	Imaging Mass Cytometry
LNs	Lymph Nodes
ECM	Extracellular matrix
MMPs	Matrix metalloproteinases
PG-SGA	Patient-Generated Subjective Global Assessment
HistoCAT	Histology Topography Cytometry Analysis Toolbox
ROI	Region of Interest
